# Investigation of Torque Teno Virus Loads as a Marker of Immunosuppression in Pediatric Chronic Renal Failure: A Pilot Study

**DOI:** 10.1111/petr.70491

**Published:** 2026-10-26

**Authors:** Ellen Kelly, Atif Awan, Dermot Wildes, Cillian F. De Gascun, Richard J. Drew, Alessandra Heggenstaller, Michael Riordan, Jaythoon Hassan

**Affiliations:** ^1^ National Virus Reference Laboratory University College Dublin Dublin Ireland; ^2^ Children's Health Ireland at Temple Street Dublin Ireland; ^3^ Rotunda Hospital Dublin Ireland; ^4^ Royal College of Surgeons in Ireland Dublin Ireland

**Keywords:** immunosuppression, kidney, pediatric, Torque Teno Virus, transplant, viral infection

## Abstract

**Background:**

Torque Teno Virus (TTV), family *Anelloviridae*, is a small, non‐enveloped, single‐stranded DNA virus that has not been linked with any specific human illness but nevertheless constitutes a major component of the human virome. An association between TTV loads and the immune status of the host with reference to other viral infections, immunosuppression therapy, and acute rejection is reported in adult and pediatric kidney transplant recipients. We sought to investigate the feasibility of TTV as a surrogate marker of the degree of immunosuppression in pediatric kidney transplant patients.

**Methods:**

185 children attending the Nephrology clinic in Children's Health Ireland at Temple Street were studied over a four‐year period from September 2019 until November 2023, ranging from 0 to 17 years of age. Patients were enrolled from pre‐transplant undergoing kidney replacement therapy (*n* = 12), from post‐transplant (*n* = 124) groups, and from patients with chronic renal failure (*n* = 20) (without kidney replacement therapy), all of which were compared to controls (*n* = 35). TTV loads were quantified using a standardized TTV R‐gene kit.

**Results:**

TTV loads were significantly higher compared to controls from 3 months post‐transplantation onward (*p* < 0.001), with the greatest increase observed between 3 and 6 months post‐transplantation. Immunosuppression exerted a significant independent effect on TTV loads after adjusting for age (*p* < 0.001). Overall, females exhibited significantly higher TTV loads than males (*p =* 0.01). Furthermore, a significant interaction was observed between patient group and gender (*p* < 0.001).

**Conclusion:**

These findings suggest that both immunosuppressive status and gender‐related factors contribute to TTV kinetics after transplantation and should be considered when interpreting TTV as a biomarker of immune function. Larger studies are required to confirm our findings.

## Graphical abstract

TTV is a promising marker to assess immune competence in a cohort of pediatric renal transplant recipients. Based on our data, immunosuppressive status and gender‐related factors affect TTV kinetics after transplantation and should be considered when interpreting TTV as a biomarker of immune function.

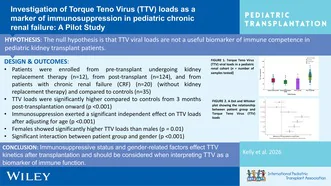

**Abbreviations**
ABMR: antibody mediated rejection
BKPyV: BK polyomavirus
CKD: chronic kidney disease
CMV: cytomegalovirus
CRF: chronic renal failure
EBV: Epstein–Barr virus
IS: immunosuppression
KTX: kidney transplant
PCR: polymerase chain reaction
QS: quantification standard
ssDNA: single‐stranded DNA
TTV: Torque Teno Virus
Tx: transplant

## 1 Introduction

Long‐term kidney function and survival after kidney transplantation rely on suitable immunosuppressive treatment to prevent organ rejection, balanced carefully with the risk of developing opportunistic infections. Therapeutic drug monitoring comprises a routine part of post‐transplant care but is limited to the individual drug in question. Current biomarkers such as lymphocyte subset analysis and neutrophil function assays lack sensitivity and specificity and fail to identify slight changes in immune response. Emerging literature has suggested a potential role for Torque Teno Virus (TTV) load as a potential surrogate marker for a patient's overall degree of immunosuppression.

The human microbiome is comprised of a wide range of highly diverse micro‐organisms. While extensive research has focused on bacterial communities, little is known about the viral populations that constitute the human microbiome and their role in immunity. Most human host tissues are colonized by members of *Anelloviridae*, a diverse family of non‐enveloped, circular, single‐stranded DNA (ssDNA) eukaryotic viruses [1]. Their ubiquity and high degree of genomic heterogeneity are well documented, with most infections affecting healthy individuals [2]. TTV is a non‐enveloped virus comprising ssDNA of negative polarity, and a guanosine cytosine (GC)‐rich region ranging from 3.7 to 3.9 kb in size [3]. Based on structural differences, there are five TTV genotypes and over 30 subtypes, with genotype 3 containing the largest number of isolates to date [4] although none have been linked to any specific human illness [5, 6]. Horizontal TTV transmission from the infected organism can occur via several routes including parenteral, sexual, and fecal‐oral; vertical transmission from mother to child has also been reported [7].

Transient changes in TTV load following iatrogenic modification of immunity suggest the virus is a plausible marker of immune function. Indeed, fluctuations in TTV load in blood are directly associated with the amount and type of immunosuppressive medications metabolized by a transplant recipient and are known to have an indirect association with graft function [8, 9, 10].

A factor to consider when assessing its utility in clinical practice is that replication of this virus is also likely to occur in an immune cell type. It has been proposed that T‐lymphocytes are the primary site for TTV replication, demonstrated by Focosi et al. [11] whereby TTV load decreased significantly following T‐cell depleting treatment with anti‐thymocyte globulin. TTV load is also 100 times higher in whole blood compared to plasma because of the presence of lymphocytes, further supporting this proposition [12]. Host antiviral responses clear > 90% of TTV infection [13] allowing TTV levels to be kept under control; however, viral persistence remains [14, 15] explaining the high prevalence in human populations, including healthy individuals.

Characterized by life‐long infection, TTV‐based guidance of immunosuppression therapy could improve risk stratification in transplant patients on an individual level, allowing for long‐term patient and graft survival. However, implementation of TTV quantification in post‐transplant clinical care would require the use of standardized diagnostic assays such as quantitative polymerase chain reaction (qPCR), as well as an accurate method for diagnosis in clinical samples.

An association between TTV plasma viral load and the immune status of the host with reference to viral infection [16, 17, 18], immunosuppression (IS) [10] and acute rejection [19, 20] has been reported in adult and pediatric kidney transplant recipients. High TTV loads and high immunosuppression are associated with a risk of infection. In contrast, the converse is associated with a risk of rejection. The CD3+ T‐receptor is essential for T‐lymphocyte activation and is therefore the main target for immunosuppressant therapies. Most investigations to date have focused on adult transplantations and report a negative association with numbers of CD4+, CD3+, CD8+ T cells, and TTV load, up to three months post‐transplant. This may be explained by partial re‐population of T‐lymphocytes, different dose regimens, multiple drug combinations, or stimulation immunosenescence by drugs such as ATG, which correlates with higher TTV loads within the first 6 months post‐transplant [21]. T‐cell depletion following induction IS appears to have short‐term effects on viral kinetics, without affecting long‐term viral replication [17]. Continued IS could trigger a widening of host cell range for TTV and/or facilitate increased replication of these newly infected cells replacing the loss of T‐cell infected cells [22].

Infections are the leading cause of death post‐transplant [23]. BK polyomavirus (BKPyV) is the main cause of polyomavirus‐associated nephropathy (BKVAN) post‐kidney transplant [24, 25]. In kidney transplant recipients, more than half of those with BK viruria will develop BK DNAemia in plasma roughly 2–6 weeks later [26, 27]. Without prophylaxis, cytomegalovirus (CMV) infection can occur in 20% of kidney transplant recipients leading to invasive disease and mortality if not treated [28]. Furthermore, a higher TTV load was also observed in patients co‐infected with CMV and Epstein–Barr virus (EBV) [21]. EBV viremia was found to be more common in kidney transplant recipients experiencing at least one opportunistic infection such as zoster or aspergillosis [29]. The association between TTV levels and graft rejection was first reported by Schiemann et al. [19]. The authors found that kidney transplant (KTX) patients with late subclinical antibody mediated rejection (ABMR) showed lower TTV levels in comparison to patients without ABMR. Moreover, with every TTV log increase, the risk of rejection decreased by an odds ratio of 0.91. The aim of this study was to prospectively follow pediatric renal transplant patients and to analyze TTV load across different stages of chronic renal failure (CRF).

## 2 Materials and Methods

### 2.1 Patients and Samples

This research project is both a “pilot study” and “method development” study; therefore, a power calculation is not required. The present study sought to prospectively describe TTV load dynamics in a cohort of chronic kidney disease (CKD) patients through to their post‐transplant care. Children attending the Nephrology clinic at Children's Health Ireland at Temple Street, were recruited for the study.

185 patients contributed data to the study with a total of 233 samples tested (Figure S1). Patients were included in one of the following cohorts: pre‐transplant; 0–3 months post‐transplant; 3–6 months post‐transplant; 6–12 months post‐transplant; 1‐year post‐transplant; > 2 years post‐transplant; CRF and healthy controls. Samples from patients more than two years post‐transplant were combined into a single cohort due to the small number of samples further out post‐transplant. Overall, 152 patients provided only one sample while 33 patients provided more than one sample. If patients provided more than one sample within a group, then these samples were averaged so that each patient only provided one measurement per group. For example, if a patient provided two samples at the pre‐transplant stage, then the mean of these two samples was included in the analysis. Overall, a total of 15 patients provided samples in more than one group. These samples were retained in the analysis, and statistical models were used which account for this clustering within patients. Eleven of these patients contributed data to two groups while four patients contributed data to three groups. The number of patients and samples in each category is shown in Table 1. Further details regarding the distribution of pre‐ and post‐transplant samples and the handling of repeated measurements per patient are provided in Table S1. For those undergoing kidney transplant, the standard immunosuppressive regimen consisted of basiliximab induction, tacrolimus, prednisolone, azathioprine, or mycophenolate. All post‐transplant patients were on maintenance immunosuppression according to our transplant guidelines at the time of sampling. Our standard dosing protocol was as follows: azathioprine 1–2 mg per kg to a maximum of 75 mg or mycophenolate mofetil 600 mg/m^2^ twice daily, tacrolimus as per trough targets and 5 mg of prednisolone on alternate days. Target tacrolimus trough levels in plasma were 10–12 ng/mL for the first three months after transplant, 7–10 ng/mL for the three‐six‐month post‐transplant period and 5–8 ng/mL after the first year. Mycophenolate and azathioprine were stopped when a patient who had detectable EBV DNA developed clinical symptoms, or when the viral load increased to more than log 10^5^ copies/mL.

**TABLE 1:** Distribution of patients and plasma samples in the study cohort.

	Number of patients (*n*)	Number of plasma samples (*n*)
Pre‐transplant	12	12
Post‐transplant 0–3 months	18	18
Post‐transplant 3–6 months	13	13
Post‐transplant 6–12 months	10	10
Post‐transplant 1 year	23	23
Post‐transplant > 2 years	73	73
Chronic renal failure	20	20
Control cohort	35	35

*Note:* The total number of samples tested was 233. The summed sample numbers across the study groups are lower than the total, as some patients had multiple samples within a group. If patients provided more than one sample within a group, then these samples were averaged so that each patient only provided one measurement per group. The summed patient numbers across the individual study groups exceed the total number enrolled because some patients contributed samples to more than one group (e.g., pre‐transplant and post‐transplant).

Ethical approval for the study was obtained from the Research Ethics Committee in Children's Health Ireland at Temple Street. Ethical exemption was obtained from the University College Dublin Research Ethics committee (LS‐E‐22‐13‐Kelly‐Hassan).

### 2.2 Nucleic Acid Extraction

A primary sample handler (Roche, Switzerland) was used to transfer 200 μL of plasma to a processing cartridge for nucleic acid extraction on the MagNA Pure 96 (Roche, Switzerland). Eluates of 100 μL were obtained following extraction. An internal control was added to all samples (10 μL) on the MagNA Pure 96, extracted, and amplified to check the efficacy of the extraction and detect the presence of possible inhibitors. A negative control (molecular grade water) was also extracted and amplified at the same time to obtain a reference per extraction run.

### 2.3 TTV Load Quantification

Following extraction, sample eluates (10 μL) and PCR master mix reagents (15 μL) were added to a 96 well Taqman plate using the Qiagility liquid handler system (Qiagen, Germany). The plate was then loaded onto the Applied Biosystems 7500 Taqman PCR instruments (Thermo Fisher, US). The TTV R‐GENE kit from Biomerieux (France) is CE‐marked and utilizes real‐time PCR technology to detect and quantify TTV in whole blood and plasma samples after extraction of the viral DNA. Ready‐to‐use amplification master mix facilitates amplification of a 128‐bp fragment in the 5′‐untranslated region of the genome. The use of the standard range supplied with the kit is imperative for sample quantification. The four quantification standards (QS1: 5000 copies/μL, QS2: 500 copies/μL, QS3: 50 copies/μL, QS4: 5 copies/μL) were used to produce a standard curve for each experiment on the basis of which the viral load of the target was calculated. The range of quantification is included between 250 copies/mL and 1 × 10^9^ copies/mL (i.e., between 2.4 log_10_ and 9.0 log_10_ copies/mL).

### 2.4 CMV, EBV, and BKPyV Viral Load Quantification

CMV, EBV, and BKPyV viral loads were quantified using TaqMan real‐time PCR assays. The Artus CMV and EBV TM PCR kits (Qiagen, Germany) are in vitro tests for the quantitation of Human CMV and EBV DNA using the ABI Prism (Taqman) 7500 Analyzer (Thermo Fisher, US). Similarly, the RealStar BKV PCR Kit (Altona, Germany) is an in vitro diagnostic test based on real‐time PCR technology for the detection and quantification of BKPyV specific DNA.

### 2.5 Clinical Chemistry

Creatinine levels were measured as part of routine testing in the biochemistry laboratory in Temple Street.

### 2.6 Statistical Analysis

Statistical analysis was performed using R version 4.5.1 [30]. Differences were considered significant at *p* < 0.05. The primary research question is whether TTV loads are a useful biomarker of immune competence in pediatric kidney transplant patients. The effect of patient specific characteristics on TTV levels was examined using linear regression models with cluster‐robust sandwich standard errors, clustered by patient ID, to account for within‐patient correlation arising from repeated measures. Patient group was included as a covariate in all models, as a small subset of patients contributed observations across multiple disease stages. TTV‐negative samples were assigned a value of 1 copy/mL prior to log_10_ transformation; sensitivity analysis excluding these samples yielded consistent results. Means and standard deviations are reported for individual variables. Dot and whisker plots are used to present the results from regression models alongside coefficient estimates and *p*‐values.

## 3 Results

### 3.1 Characteristics of the Study Population

The demographics and characteristics of the patients are described in Table 1A. Pre‐transplant patients were defined as those with CKD who were receiving kidney replacement therapy at the time of sampling whereas the CKD group were not receiving kidney replacement therapy at the time of TTV sampling. TTV DNA was detected in 85% of patients with CKD (17 of 20). Among pre‐transplant patients, 58% were TTV DNA positive (7 of 12). 10 patients were receiving dialysis, although the duration of dialysis was not recorded. Of these, seven were receiving peritoneal dialysis (PD) and three were receiving hemodialysis (HD). The remaining two patients underwent pre‐emptive transplantation. Only six of the twelve pre‐transplant patients had corresponding post‐transplant samples tested. A further 22 post‐transplant patients provided multiple samples (Table S1).

**TABLE 1A:** Characteristics of patient groups in the study.

Patient demographics and clinical characteristics	Controls	Chronic renal failure	Pre‐Tx	Post‐Tx	Transplant group (pre and post‐Tx combined)
Sex, *n* (%)					
Male	23 (66)	16 (80)	5 (42)	57 (46)	—
Female	12 (34)	4 (20)	7 (58)	67 (54)	—
Primary kidney disease, *n* (%)					
CAKUT (Congenital anomalies of the kidney and urinary tract)	—	—	—	—	81 (62)
Hereditary kidney disease	—	—	—	—	26 (20)
Nephrotic syndrome	—	—	—	—	10 (8)
Hemolytic uremic syndrome (HUS)	—	—	—	—	7 (5)
Wilm's tumor	—	—	—	—	4 (3)
Cystinosis	—	—	—	—	1 (1)
Post septic shock (leading to acute kidney injury)	—	—	—	—	1 (1)
Immunosuppressive therapy, *n* (%)					
Basiliximab (induction)	—	—	—	124 (100)	—
Tacrolimus (maintenance)	—	—	—	124 (100)	—
Prednisolone	—	—	—	124 (100)	—
Azathioprine/Mycophenolate	—	—	—	124 (100)	—
Azathioprine/Mycophenolate adjusted (EBV viral load > log 5 copies/mL)^a^	—	—	—	7/79 (9)	—
Maintained azathioprine/Mycophenolate (EBV viral load < log 5 copies/mL)^a^	—	—	—	72/79 (91)	—
Dialysis					
Hemodialysis (HD)	—	—	3 (25)	—	—
Peritoneal dialysis (PD)	—	—	7 (58)	—	—
Continuous metrics, median (IQR)					
Age at TTV sample collection (years)	12 (8–13)	12 (8–15)	8 (4–13)	11 (8–14)	—
Age at KTX (years)	—	—	—	6 (4–9)	—
Creatinine (μmol/L)	51 (46–63)	287 (98–414)	444 (272–679)	75 (57–108)	

*Note:* A total of 185 patients were enrolled, the majority being male and with a median age of 11 years. The most common underlying disease in the transplant cohort was CAKUT (congenital anomalies of the kidney and urinary tract). Pre‐transplant patients were defined as individuals with chronic kidney disease (CKD) who were receiving kidney replacement therapy at the time of sampling, whereas the CRF group comprised patients who were not receiving kidney replacement therapy at the time of TTV sampling. Among the pre‐transplant patients, 10 were receiving dialysis, although the duration of dialysis was not recorded. Of these, seven were receiving peritoneal dialysis (PD) and three were receiving hemodialysis (HD). The remaining two patients underwent pre‐emptive transplantation. The standard immunosuppressive regimen for all patients consisted of basiliximab induction, tacrolimus, prednisolone, azathioprine, or mycophenolate. Target tacrolimus trough levels in plasma were 10–12 ng/mL for the first three months after transplant, 7–10 ng/mL for the three‐to‐six‐month post‐transplant period, and 5–8 ng/mL after the first year.

Abbreviations: TTV, Torque Teno Virus; Tx, transplant.

^a^ EBV viral load data was available for a subset of post‐transplant patients (*n* = 79). Of these, 72 (91%) remained on AZA/MMF, while 7 (9%) had their therapy adjusted due to an EBV viral load > 5 log copies/mL.

The timing of plasma collection relative to transplantation varied between patients. At the time of sampling, some individuals were only days post‐transplant, whereas others were one to two months post‐transplant, and some had undergone transplantation several years earlier. To account for this variability, post‐transplant patients were stratified according to the interval between transplantation and TTV sample collection into the following categories: 0–3 months, 3–6 months, 6–12 months, 1 year post‐transplant and > 2 years post‐transplant. Overall, TTV DNA was detected in 94% of post‐transplant patients (117 of 124). Control patients were recruited from immunocompetent members of the general nephrology outpatient cohort. They were defined as children naïve to immunosuppression who attended the nephrology outpatient clinic with no underlying immunodeficiency. 92% of these patients were found to be positive for TTV DNA (32 of 35).

### 3.2 TTV Load Kinetics in the Patient Cohorts

The pre‐transplant group, control cohort and CRF group had similar TTV loads (mean ± SD log_10_copies/mL: 2.2 ± 2.1 vs. 3.0 ± 1.4 vs. 2.6 ± 1.4) (Figure 1). During sample analysis, TTV data from the first‐year post‐transplantation was stratified into 0–3, 3–6, and 6–12‐month intervals to address temporality. These intervals were analyzed separately and compared with TTV data obtained beyond the first post‐transplant year. TTV levels increase from baseline levels up to months 3–6 post‐transplant (mean ± SD log_10_copies/mL: 5.7 ± 1.7) (Figure 2). In addition, TTV levels at 6–12 months and beyond the first post‐transplant year were higher compared to pre‐transplant values (mean ± SD log_10_copies/mL: 5.2 ± 1.1 vs. 4.7 ± 1.6 vs. 2.2 ± 2.1). Figure 2A shows there is no discernible difference in TTV levels in the CRF or pre‐transplant group compared with controls, but TTV levels are higher post‐transplant. TTV load seems to gradually lower the longer it has been since the patient received a transplant, although the confidence intervals for the post‐transplant groups are overlapping so it is difficult to be confident in this downward trend. TTV loads were significantly higher than in controls from 3 months post‐transplantation onward (*p* < 0.001), with the greatest increase observed between 3 and 6 months post‐transplantation. The effect of each treatment group on TTV relative to the control cohort is shown in Table S2.

**FIGURE 1:**
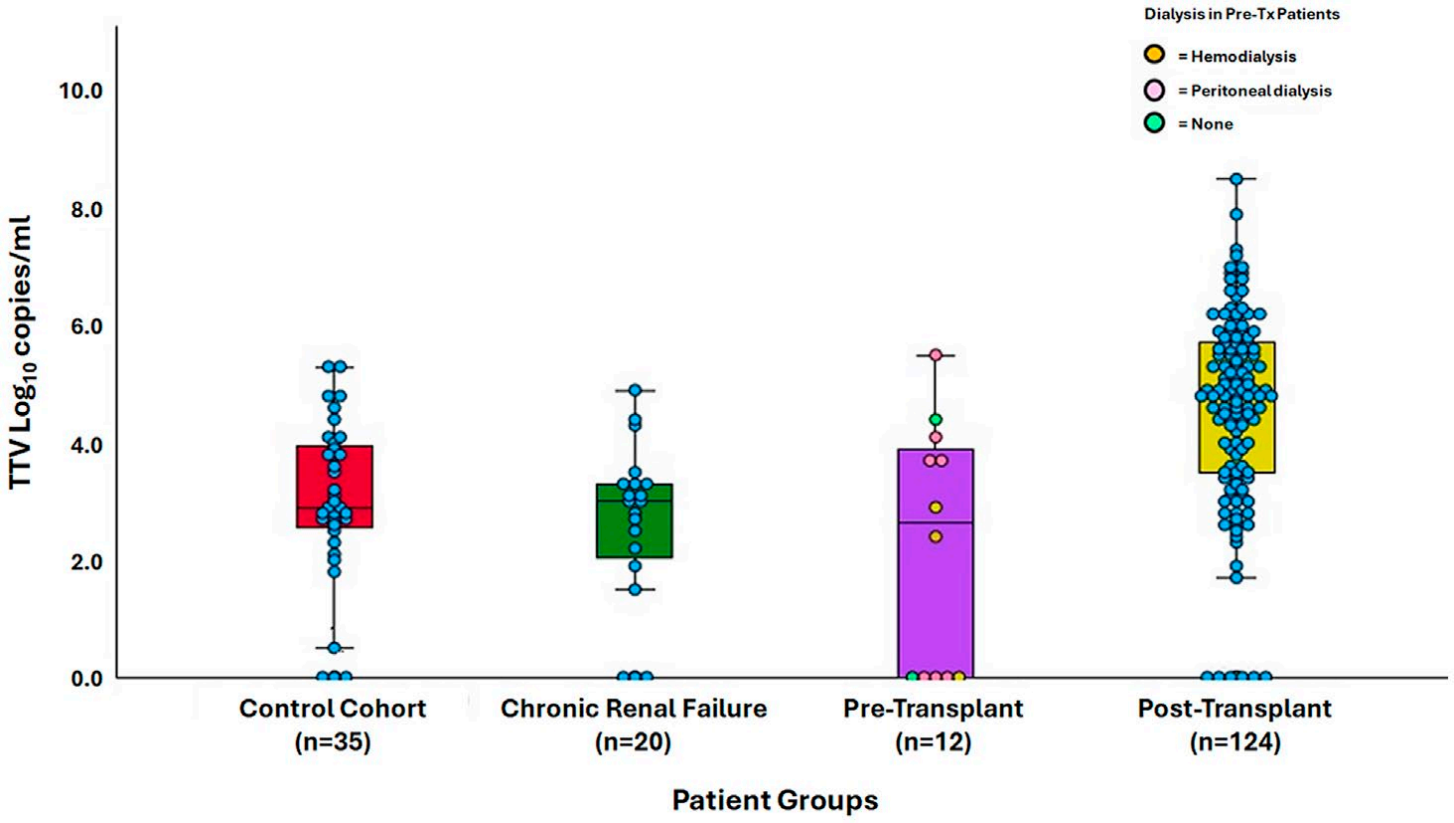
All Torque Teno Virus (TTV) loads in a pediatric renal cohort (*n* = 191). The pre‐transplant group, control cohort, and chronic renal failure group have similar TTV loads. Post‐transplant patients have higher TTV loads in comparison to the other patient groups. Seven pre‐transplant patients were receiving peritoneal dialysis (PD), and three were receiving hemodialysis (HD). The remaining two patients underwent pre‐emptive transplantation. Mean values are shown where *n* = number of samples tested. For patients who had more than one sample tested within their group, an average TTV load was calculated. Tx, transplant.

**FIGURE 2:**
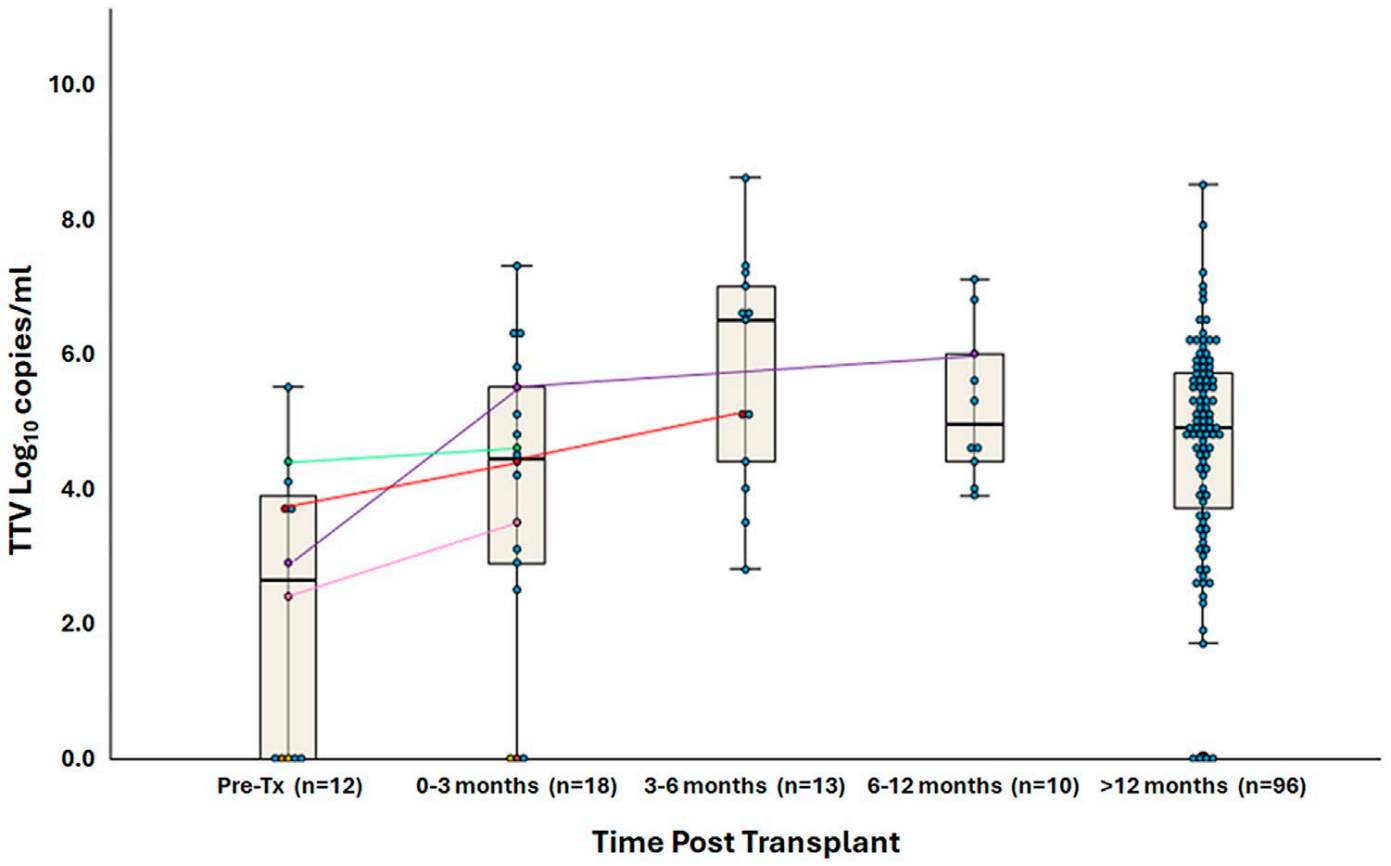
Beeswarm‐Boxplot of Torque Teno Virus (TTV) load in a pediatric cohort in relation to time after renal allograft transplantation. TTV levels increase from baseline levels up to months 3–6 post‐transplant. TTV levels are also elevated between 0–3 and 3–6 months post‐transplant. In addition, TTV levels at 6–12 months and beyond the first post‐transplant year were higher compared to pre‐transplant values. A decrease in TTV loads is observed beyond 12 months post‐transplant compared with the 3–6‐month interval. Overlap between pre‐ and post‐transplant samples could only be retrospectively inferred from laboratory identifiers. Six of twelve pre‐transplant patients had identifiable corresponding post‐transplant samples. Serial samples from these patients have been denoted by color and connecting lines to show individual trajectories. A further 22 patients contributed multiple post‐transplant samples. *n* = number of samples tested. For patients who had more than one sample tested within their group, an average TTV load was calculated. Tx, transplant.

**FIGURE 2A:**
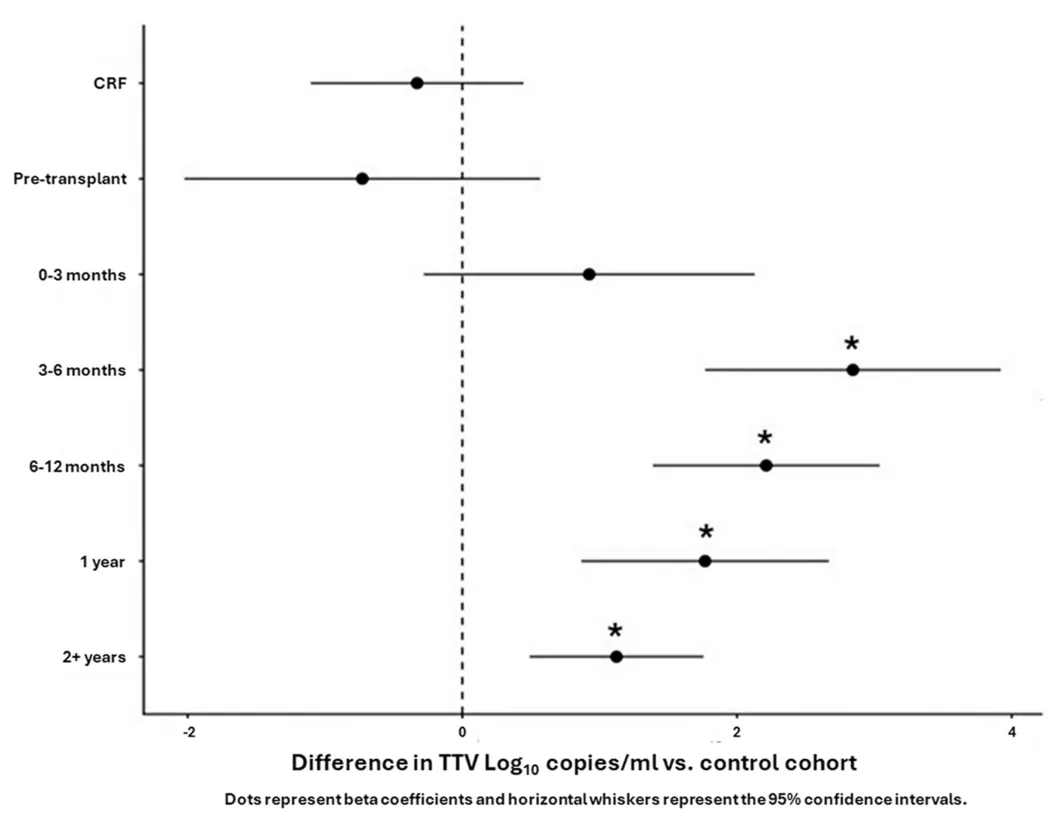
A dot and whisker plot showing the relationship between patient group and Torque Teno Virus (TTV) loads. A linear regression with sandwich standard errors was used to examine differences in TTV by patient group. The outcome was TTV log_10_ copies/mL. Patient group was dummy coded and added as a predictor with the control cohort as the reference group so that average TTV levels in each group were compared with those in the control cohort. The dots represent beta coefficients and the horizontal lines represent 95% confidence intervals. There is no difference in TTV levels in the CRF or pre‐transplant group compared with the controls. TTV levels are significantly higher 3–6 months post‐transplant onward and gradually stabilize with longer time since transplantation. *p ≤ 0.001.

### 3.3 Age and Gender Associations With TTV Loads

Linear regression analysis was used to examine the association between age and TTV in patients with and without IS. Mean age in those receiving IS was 10.45 years (SD = 3.67) and was 10.49 (SD = 3.91) for those not on IS (Figure 3). In a model with age and immunosuppression predicting TTV there was a significant effect of IS therapy [*B* = 1.57, SE = 0.24, *p* < 0.001] but not age (*p* = 0.92). When an interaction between age and immunosuppression was added to the model, the association between age and TTV in patients with and without IS was not significant (*p* = 0.24). Additionally, when patient group (which captures time post‐transplant) was included in the model in place of IS status, age remained non‐significant (*β* = −0.001, SE = 0.040, *p* = 0.98). As a further sensitivity analysis within immunosuppressed patients only, we tested whether the effect of age on TTV varied by time post‐transplant. The interaction between age and post‐transplant timepoint was not significant (*F*(4, 127) = 0.93, *p* = 0.45).

**FIGURE 3:**
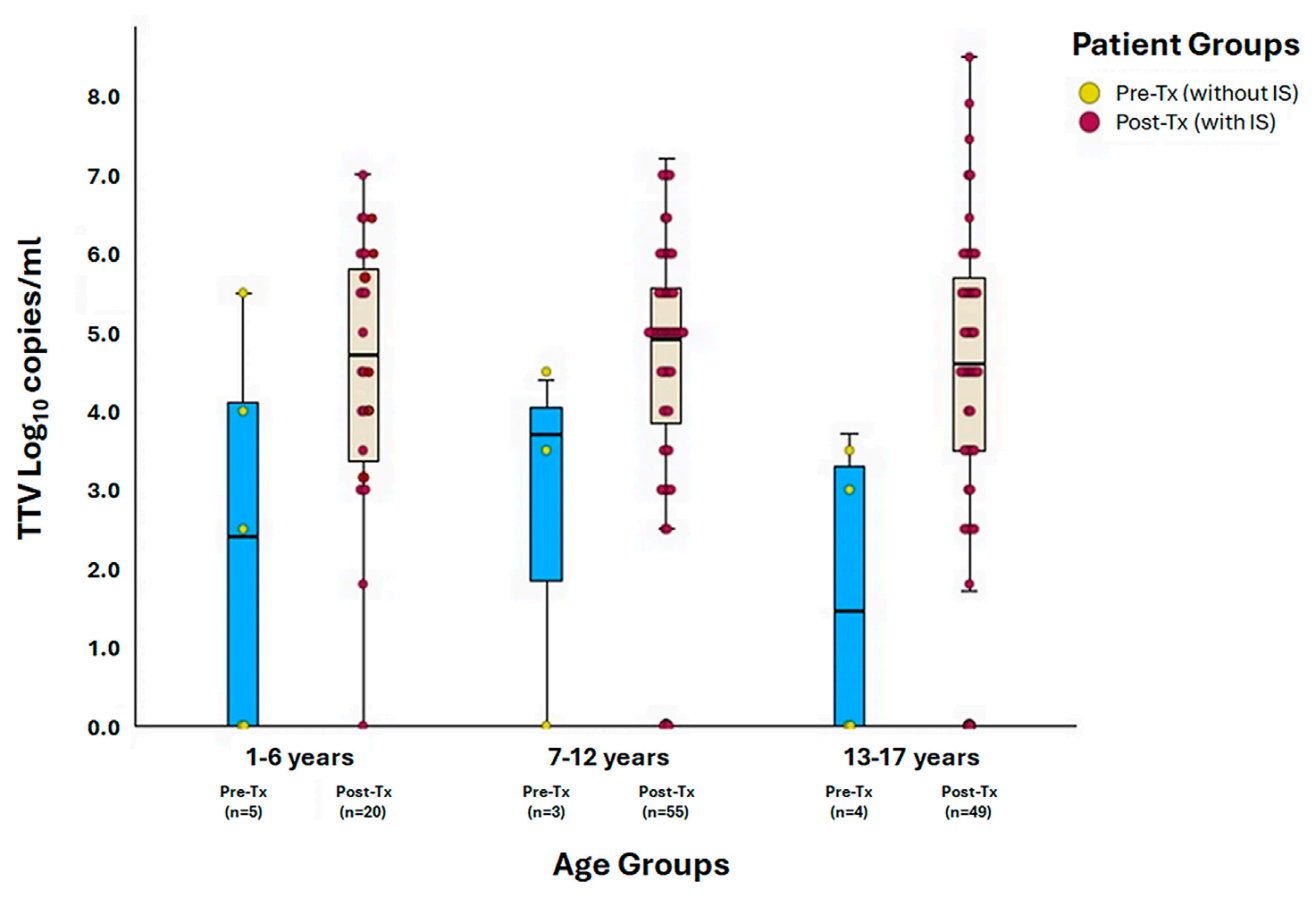
Boxplot comparing Torque Teno Virus (TTV) loads with respect to age at time of TTV sample collection in patients with and without immunosuppression. Across all age groups, post‐transplant patients exhibited higher TTV viral loads than pre‐transplant patients. *n* = number of samples tested. For patients who had more than one sample tested within their age group, an average TTV load was calculated. IS, immunosuppression; Tx, transplant.

TTV prevalence ranged from 90% in 1–6‐year‐olds (27 of 30) to 91% in 7–12‐year‐olds (74 of 81) and to 92% in 13–17‐year‐old patients (68 of 74).

Overall, TTV loads were significantly higher in females compared to males (mean ± SD log_10_ copies/mL: 4.30 ± 2.06 vs. 3.53 ± 1.86, *p* = 0.01). However, in a model with patient group included, the effect of sex is no longer significant (*p* = 0.10) since female patients are overrepresented post‐transplant. Figure 3A shows a significant interaction between patient group and sex [*F*(7, 188) = 3.84, *p* < 0.001], indicating that the effect of patient group on TTV load differed between males and females. Females demonstrated markedly higher TTV levels in the early post‐transplant period, peaking at 3–6 months (mean ± SD log_10_copies/mL: 6.6 ± 1.2), while males showed a more attenuated response.

**FIGURE 3A:**
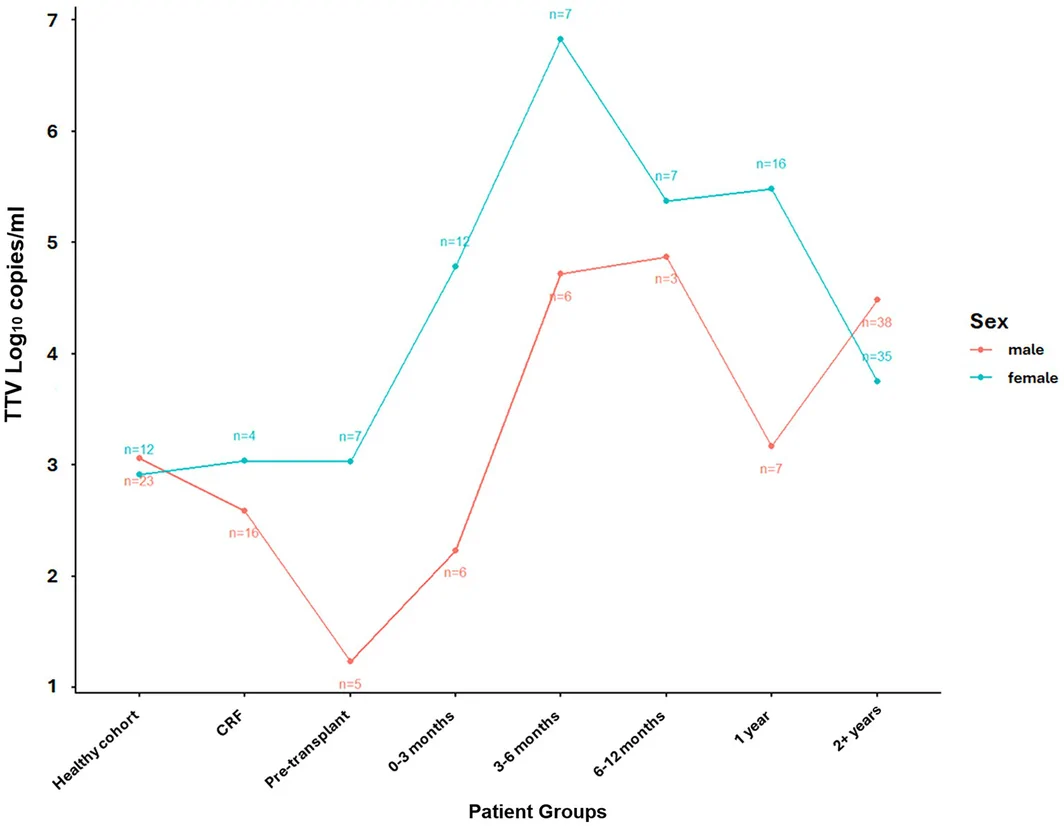
Interaction plot of mean Torque Teno Virus (TTV) levels by gender and patient group. Mean TTV levels in males and females across the patient groups are shown. A significant interaction between patient group and sex was observed (*p* < 0.001), indicating the effect of patient group on TTV levels between males and females. Females exhibited higher TTV loads during the early post‐transplant period, peaking at 3–6 months, whereas males showed a less pronounced increase.

There was no significant interaction between the effect of age and gender on TTV load (*p* = 0.57).

### 3.4 Correlation of TTV Plasma Load With CMV, EBV, and BKPyV Infection

CMV was tested for in eight pre‐transplant patients, and all were negative. BKPvV was tested for in three pre‐transplant patients, and all were negative. In the post‐transplant cohort, 78 of 124 patients had available CMV data. Of these, three patients (3.85%) had detectable CMV DNA, while the remainder were negative. Similarly, 76 of 124 post‐transplant patients had reportable BKPyV data; among them, ten (13.16%) had detectable BKPyV DNA.

EBV DNAemia was defined as a quantitative positive EBV PCR result > 10^3^ copies/mL. Although donor EBV serology was unfortunately unavailable in our dataset, pre‐transplant EBV status was assessed by blood testing prior to listing for transplantation, and a look‐back analysis was conducted on available recipient pre‐ and post‐transplant serology.

All pre‐transplant patients were asymptomatic at the time of sampling and were predominantly in the 1–6 and 7–12‐year age groups. EBV data were unavailable for 4 pre‐transplant patients. Of the remaining 8, five had detectable TTV DNA with available EBV PCR results. Of these five patients, three (60%) had EBV DNAemia, while two were EBV DNA negative. Three patients were negative for both viruses (Figure 4).

**FIGURE 4:**
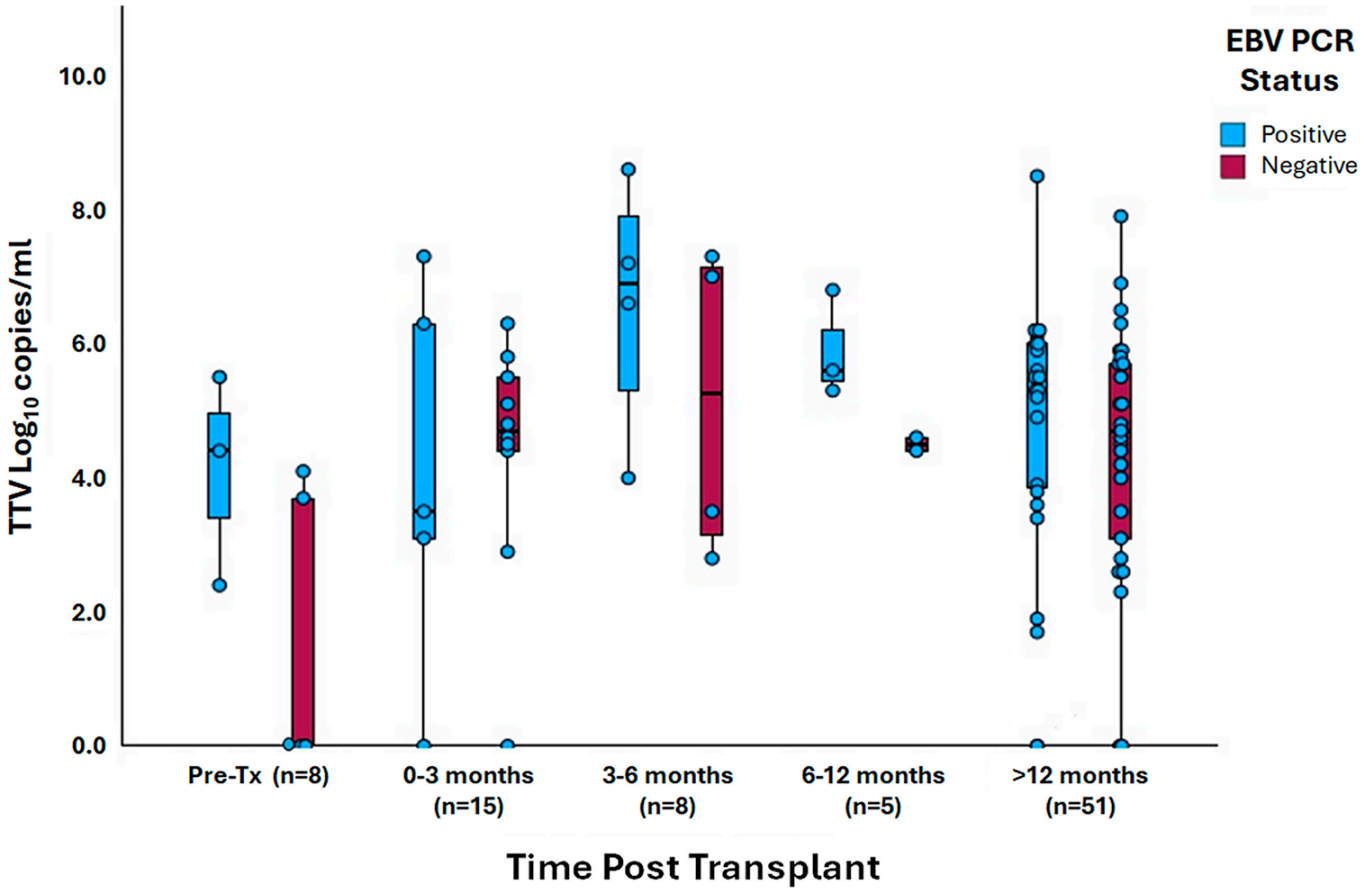
Clustered box plot of Torque Teno Virus (TTV) levels against EBV PCR status across different time points. The EBV positive group is defined based on quantitative positive EBV PCR, > 10^3^ copies/mL and the EBV negative group based on negative EBV PCR. EBV viral loads were quantified using the Artus EBV TM PCR Kit (Qiagen, Germany), which has an assay cut‐off of 500 copies/mL (2.7 log_10_ copies/mL). TTV levels increased following transplantation, with the highest values observed between 3 and 6 months post‐transplant in both EBV‐positive and EBV‐negative patients. EBV‐positive patients generally exhibited higher and more heterogeneous TTV viral loads compared with EBV‐negative patients, particularly pre‐transplant and in the early (0–6 months) post‐transplant periods. At > 12 months post‐transplant, TTV levels remained elevated in both groups. *n* = number of samples tested. Sample numbers differ from those in other graphs as EBV data were unavailable for some patients included in the cohort. For patients who had more than one sample tested, an average EBV load was calculated.

In the post‐transplant setting, EBV data were available for 79 of the 124 post‐transplant patients. Of these, 38 (48.10%) had detectable EBV DNA, while the remainder were EBV DNA negative. Serologically, most patients post‐transplant developed VCA IgG positivity while remaining EBNA IgG negative for variable periods of time.

The relationship between TTV and CMV, EBV, and BKPyV was examined using a regression model. Positivity for any of the three viruses was first examined in combination. 52 patients were positive for at least one of the three viruses while 52 were negative. There was no significant relationship between positivity for one of the three viruses and TTV (*p* = 0.10). In addition, the relationship between TTV and EBV viral load was examined in post‐transplant patients using a linear regression model with cluster‐robust sandwich standard errors controlling for time post‐transplant. EBV viral load was not significantly associated with TTV (*p* = 0.43) (Figure S2).

Assessment of TTV loads in relation to acute rejection episodes or donor‐specific antibodies in the post‐transplant cohort was beyond the scope of the present study.

### 3.5 Correlation of TTV Plasma Load With Creatinine

There was no significant effect of creatinine on TTV levels after controlling for patient group (*p* = 0.57). In patients more than 12 months after transplantation, differences in creatinine level between those who were positive and negative for EBV were examined using a Welch's *t*‐test. Mean creatinine in those who were EBV positive was 1.87 ± 0.14 log_10_ copies/mL compared with 2.02 ± 0.27 log_10_ copies/mL in those who were EBV negative. This difference was statistically significant [*t*(40.32) = 2.51, *p* = 0.016].

### 3.6 Correlation of TTV Plasma Load With Immunosuppression Therapy

The relationship between TTV loads and trough levels of IS was assessed in post‐transplant patients starting after 3 months transplantation. IS therapy consisted of Tacrolimus, Myfortic, and Prednisolone. There was no significant effect of trough levels on TTV in patients three months or more post‐transplant when controlling for time post‐transplant: 3–6 months (*n* = 13), 6–12 months (*n* = 9), and > 12 months (*n* = 92). Tacrolimus trough levels were unavailable for a subset of patients' post‐transplant. Immunosuppression levels were categorized binarily as therapeutic or subtherapeutic based on target tacrolimus trough concentrations. There was no significant difference in TTV loads in patients 3 months or more post‐transplant with subtherapeutic versus therapeutic levels of IS (*p* = 0.24).

## 4 Discussion

We conducted a prospective study of pediatric patients, profiling TTV load kinetics across pre‐ and post‐transplant, CKD, and healthy patients. The potential application of TTV viral kinetics as a biomarker of immune status has been explored in the literature in recent years. The mean TTV load in a cohort of adult healthy blood donors was reported as 2.3 ± 0.7 log copies/mL, median 2.3 log copies/mL; range 1.1–4.9 log copies/mL [11]. This is reflective of a high daily turnover generation rate of > 10 log copies/mL with > 90% cleared by the immune system [13]. The immune system plays an important role in controlling TTV infection, and for this reason, plasma viral loads tend to be higher in individuals with immune system dysfunction. This was reflected in our cohort, where there were significantly higher TTV levels in post‐transplant patients who were immunosuppressed, compared to their healthy control counterparts.

In our study, TTV loads peaked early post‐transplant and gradually declined over time. This finding agrees with Uhl et al. [10] and Schiemann et al. [19] who demonstrated lower viral loads over a longer post‐transplant time in both a pediatric renal cohort and in adult renal recipients. Similarly, Fernández‐Ruiz et al. [21] observed a gradual decrease beyond the first year. TTV loads were significantly higher than in controls from 3 months post‐transplantation onward, with the greatest increase observed between 3 and 6 months post‐transplantation. This agrees with the findings of Solis et al. [31] and Fernández‐Ruiz et al. [21] who reported an increase from baseline TTV levels to month 3, peaking between 3 and 12 months post‐transplant, in adult kidney transplant patients.

IS therapy usually results in a significant increase in TTV load, but exactly how TTV kinetics during months 1–3 post‐transplant correlate with immunosuppressive state is still unclear. Consequently, many research groups avoid assessing TTV levels during the first three months post‐transplantation. As mentioned above, most adult studies report a negative association with numbers of CD4^+^, CD3^+^, CD8^+^ T‐cells and TTV load up to 3 months post‐transplant. All transplant patients in this study received induction IS with basiliximab, while maintenance therapy consisted of tacrolimus, mycophenolate, and prednisolone. Basiliximab inhibits the activation and proliferation of T‐cells rather than causing T‐cell depletion and therefore has minimal impact on TTV load [15]. While the T‐cell number is not reduced, the ability to control TTV proliferation is reduced [15, 32]. Higher TTV loads were evident in KTX patients on tacrolimus, a calcineurin inhibitor, compared to cyclosporine, or mammalian targets of rapamycin inhibitors [10, 19]. Moreover, in children, TTV DNA levels were found to be 2‐fold higher when receiving tacrolimus [10] in agreement with the study conducted by Schiemann et al. [19]. This may explain the high viral loads seen in transplant patients during months 3–6 post‐transplant. A larger host cell range for TTV could develop with continued IS facilitating increased replication of these infected cells [22].

A high prevalence of TTV, which increases with age, has been reported in human adult populations [33]. This could be explained by the evolution of existing lineages and acquisition of new strains over time [11]. Higher TTV levels may reflect immunosenescence, which leads to increased risk of infection, frailty, and mortality in older people [33]. The oldest age group had the highest TTV prevalence overall. Interestingly, our study found a significant independent association between IS and TTV load after adjusting for age. Age was not associated with TTV load, similar to the findings of Uhl et al. [10], regardless of whether a patient was immunosuppressed or not and after adjusting for time post‐transplant. These findings support the concept that TTV is primarily a marker of overall immunosuppressive burden. TTV kinetics appear more closely related to the presence of immunosuppression rather than age‐related immunosenescence.

Evidence suggests that males and females have different immune responses to infection, with males generally more susceptible. This in part could be due to hormonal effects on the host's immune system, which are age dependent. Overall, female children in this study had significantly higher TTV loads. This contrasts with the findings of Borštnar et al. [34] who found significantly higher TTV levels in male KTX recipients. Females exhibited higher TTV loads during the early post‐transplant period, peaking at 3–6 months, whereas males showed a less pronounced increase. We observed a significant interaction between patient group and gender, meaning TTV levels change differently across patient groups in males compared with females.

A small number of patients were positive for CMV, EBV, and BKPyV in this study. For that reason, positivity for any of the three viruses was examined in combination. Our findings indicate that prior exposure to CMV, EBV, or BKPyV (when considered together) is not associated with differences in TTV levels, indicating that TTV replication appears independent of the presence of these common viral infections in this cohort. Handala et al. [35], reported a non‐significant correlation between TTV load and BKPyV viremia in their study cohort. This contrasts with Uhl et al. [10] who reported an almost significant correlation with BKPyV. Eibensteiner et al. [18] reported a robust independent association between TTV plasma load and the onset of CMV and BKPyV infections—both indicators of over‐immunosuppression—even after adjusting for important confounders such as age and time post‐transplant. Moreover, at a median of 33 days following transplantation, TTV plasma load could predict the onset of both these infections [18]. EBV infection was more prevalent in this study in comparison to the other transplant‐specific viruses. Eibensteiner et al. [18], Garbuglia et al. [36], Borkosky et al. [37] and Mallet et al. [38] all found a significant correlation between TTV and EBV viremia. This differs from our findings, which showed no association between the two variables. In our study, TTV levels increased following transplantation, with the highest values observed between 3 and 6 months post‐transplant in both EBV‐positive and EBV‐negative patients. This agrees with the findings of Cañamero et al. [39] who showed higher TTV loads during months 1–6 post renal transplant in patients who experienced other infections. EBV‐positive patients generally exhibited higher and more heterogeneous TTV viral loads compared with EBV‐negative patients, particularly pre‐transplant and in the early (0–6 months) post‐transplant periods. Children who develop positive EBV PCR have a higher TTV level at the time of transplant. It would be plausible that children who enter the transplant with a more immunosuppressed state, as demonstrated by a higher TTV level at the time of transplant, are more likely to have difficulties post‐transplant with EBV reactivation. Although pre‐ and post‐transplant EBV serology was evaluated in our cohort, donor EBV serostatus was unavailable. Post‐transplantation, the majority of patients demonstrated VCA IgG positivity while remaining EBNA IgG negative for variable durations. This delayed or absent EBNA IgG response reflects the altered humoral immune kinetics typical of immunosuppressed transplant patients. In this study, our findings demonstrated that creatinine was not associated with TTV levels after accounting for patient group. Among patients more than 12 months after transplantation, EBV‐positive patients had significantly lower creatinine levels than EBV‐negative patients, indicating a statistically significant difference in kidney function between the groups. This contrasts with previous studies which suggest that renal transplant patients with positive EBV viremia may be at higher risk for elevated serum creatinine [40]. One possible explanation is that detectable EBV reflects a greater degree of immunosuppression and is associated with a risk of infection but may protect against immune‐mediated graft injury and thus preserve renal function. Alternatively, patients with impaired graft function may have undergone immunosuppression reduction, resulting in improved viral control and lower rates of EBV detection.

The current study has some limitations common with single center pilot studies. A key limitation is the relatively small pre‐transplant cohort and the limited number of patients with serial TTV measurements following transplantation, which restrict the longitudinal assessment of TTV dynamics and reduce the statistical power. As a single‐center study, these results should therefore be validated in larger, multicenter cohorts with more comprehensive longitudinal follow‐up. Nonetheless, findings from this study show that TTV is a promising marker to assess immune competence in a cohort of pediatric renal transplant recipients and agree with previous similar reports in adult and pediatric kidney transplant patients. Based on our data, immunosuppressive status and gender‐related factors affect TTV kinetics after transplantation and should be considered when interpreting TTV as a biomarker of immune function. TTV load was not influenced by the presence of CMV, EBV, or BKPyV viral infection. Although higher TTV levels at the time of transplant could identify patients who are more likely to have difficulties post‐transplant with EBV reactivation and should be more closely monitored. In conclusion, multi‐center studies with larger patient numbers are required to evaluate the significance and relevance of these findings.

## Author Contributions

Patients were recruited for the study by Prof. Atif Awan, Dr. Michael Riordan, and Dr. Dermot Wildes. Alessandra Heggenstaller labeled and sent the samples for testing to the National Virus Reference Laboratory (NVRL). Ellen Kelly performed TTV testing, wrote, and revised the manuscript. Dr. Cillian F. De Gascun and Richard Drew reviewed the manuscript. Prof. Jaythoon Hassan was involved in overseeing all experimental procedures, ordering reagents and materials, and advising on methods and result analysis. All co‐authors reviewed the manuscript.

## Funding

This work was supported by Children's Health Foundation, Ireland (Grant RPAC19‐05).

## Conflicts of Interest

The authors declare no conflicts of interest.

## Supporting information


**Figure S1:** STROBE flow diagram outlining study population eligibility and selection.

**Figure S2:** Association between Torque Teno Virus (TTV) loads and EBV viral infection.

**Table S1:** Pre‐ and post‐transplant sample distribution and handling of repeated measurements per patient.
**Table S2:** The effect of patient group on TTV load relative to control cohort.

## Data Availability

The data that support the findings of this study are available from the corresponding author upon reasonable request.
