# Supplementary material for: Investigation of Torque Teno Virus Loads as a Marker of Immunosuppression in Pediatric Chronic Renal Failure: A Pilot Study
Source: Pediatr Transplant. 2026 Oct 26;30(11):e70491. doi: 10.1111/petr.70491 (PMC13700081; doi:10.1111/petr.70491)
Supplement: Supplementary file 1 [file PETR-30-e70491-s001.docx]

**Supplementary Figure 2:** Association between Torque Teno Virus (TTV) loads and EBV viral infection

Scatterplot illustrating the relationship between TTV load (log₁₀ copies/mL) and viral load of Epstein–Barr virus (EBV) in post-transplant samples. No clear correlation between TTV load and EBV viral load was observed. The scatter plot does not show negative samples for EBV. Red data points indicate samples with viral loads detected below the assay limit of quantification (EBV: <2.7 log₁₀ copies/mL).
