# Supplementary material for: Investigation of Torque Teno Virus Loads as a Marker of Immunosuppression in Pediatric Chronic Renal Failure: A Pilot Study
Source: Pediatr Transplant. 2026 Oct 26;30(11):e70491. doi: 10.1111/petr.70491 (PMC13700081; doi:10.1111/petr.70491)
Supplement: Supplementary file 2 [file PETR-30-e70491-s002.docx]

**Supplementary Figure 1: STROBE Flow Diagram Outlining Study Population Eligibility and Selection**

Assessed in the Nephrology clinic in Children’s Health Ireland at Temple Street

1 of 1

Eligible participants enrolled (n=204)

Pre –Tx (n=12)

Post –Tx (n=124)

**Eligibility**

**Enrollment**

**Data Analysis**

**Follow-Up**

Participants with completed baseline assessment (n=185)

Final analytic cohort included in analysis (n=191)

Control Cohort (n=35)

Chronic Renal Failure (n=20)

Four-year period from September 2019 - November 2023 (n=204)

Healthy Controls excluded during follow up (n=19) – insufficient laboratory data

Participant screening, enrolment, group allocation, and final sample inclusion across study stages are shown. Specific reasons for participant exclusion are detailed within the diagram. The summed patient numbers across the individual study groups (n=191) exceed the total number enrolled (n=185) because some patients contributed samples to more than one group (e.g. pre-transplant and post-transplant). If patients provided more than one sample within a group, then these samples were averaged so that each patient only provided one measurement per group.
