# Supplementary material for: Investigation of Torque Teno Virus Loads as a Marker of Immunosuppression in Pediatric Chronic Renal Failure: A Pilot Study
Source: Pediatr Transplant. 2026 Oct 26;30(11):e70491. doi: 10.1111/petr.70491 (PMC13700081; doi:10.1111/petr.70491)
Supplement: Supplementary file 3 [file PETR-30-e70491-s003.docx]

**Supplementary Table S1:** Pre- and Post-Transplant Sample Distribution and Handling of Repeated Measurements per Patient

| Patients | Time of Sampling (Months) | TTV Load (log_10_ copies/ml) | Mean TTV load (log_10_ copies/ml ±SD) |
| --- | --- | --- | --- |
| 1 A  1 B  1 C  1 D  1 E | Pre-Tx  0-3 months post-tx  0-3 months post-tx  0-3 months post-tx  3-6 months post-tx | 3.7  3.6  3.4  5.6  5.1 | 4.2 ± 1.2 |
| 2 A  2 B  2 C  2 D | Pre-Tx  Pre-Tx  0-3 months post-tx  0-3 months post-tx | 3.8  5.0  3.5  5.2 | Not calculated |
| 3 A  3 B | >2 years post-tx  >2 years post-tx | 5.1  2.7 | Not calculated |
| 4A  4B | >2 years post-tx  >2 years post-tx | 5.0  5.1 | 5.1± 0.7 |
| 5A  5B | >2 years post-tx  >2 years post-tx | 5.2  6.6 | 5.9±1.0 |
| 6A  6B  6C | Pre-Tx  Pre-Tx  0-3 months post-tx | Negative  Negative  Negative | Negative |
| 7A  7B  7C  7D  7E | 0-3 months post-tx  0-3 months post-tx  0-3 months post-tx  3-6 months post-tx  6-12 months post -tx | 6.6  5.8  6.7  6.6  7.1 | 6.4 ± 0.5 |
| 8A  8B  8C | 0-3 months post-tx  3-6 months post-tx  6-12 months post -tx | Negative  5.1  4.6 | N/A |
| 9A  9B | >2 years post-tx  >2 years post-tx | 3.2  1.9 | 2.6 ±0.9 |
| 10A  10B | 3-6 months post-tx  1-year post-tx | 7.8  6.5 | N/A |
| 11A  11B  11C | Pre-Tx  0-3 months post-tx  6-12 months post -tx | 2.9  5.8  6.0 | N/A |
| 12A  12B  12C | >2 years post-tx  >2 years post-tx  >2 years post-tx | 4.8  4.3  4.8 | 4.6 ±0.4 |
| 13A  13B | >2 years post-tx  >2 years post-tx | 5.0  4.9 | 5.0± 0.1 |
| 14A  14B | Pre-Tx  0-3 months post-tx | Negative  Negative | N/A |
| 15A  15B | 3-6 months post-tx  3-6 months post-tx | 3.6  3.3 | 3.5± 0.2 |
| 16A  16B | 1-year post-tx  1-year post-tx | 8.6  8.4 | 8.5 ±0.1 |
| 17A  17B | 0-3 months post-tx  3-6 months post-tx | 2.9  4.4 | N/A |
| 18A  18B | 3-6 months post-tx  6-12 months post -tx | 4.1  3.9 | N/A |
| 19A  19B | 1-year post-tx  1-year post-tx | 6.6  5.4 | 6.0± 0.8 |
| 20A  20B | 3-6 months post-tx  6-12 months post -tx | 7.3  5.6 | N/A |
| 21A  21B | 1-year post-tx  1-year post-tx | 4.5  4.8 | 4.7± 0.2 |
| 22A  22B | >2 years post-tx  >2 years post-tx | 4.4  4.7 | 4.6 ±0.2 |
| 23A  23B  23C | Pre-Tx  0-3 months post-tx  0-3 months post-tx | 2.4  3.4  3.5 | 3.5 ± 0.1 |
| 24A  24B  24C | 0-3 months post-tx  0-3 months post-tx  3-6 months post-tx | 4.2  6.7  8.6 | Not calculated |
| 25A  25B  25C | 0-3 months post-tx  0-3 months post-tx  3-6 months post-tx | 4.7  5.4  6.5 | 5.1± 0.5 |
| 26A  26B | Pre-tx  Pre-tx | 5.0  6.0 | 5.5 ±0.7 |
| 27A  27B | 0-3 months post-tx  3-6 months post-tx | 3.1  6.6 | N/A |
| 28A  28B | 1-year post-tx  1-year post-tx | 4.7  4.8 | 4.8 ±0.1 |
| 29A  29B | 1-year post-tx  1-year post-tx | 7.0  7.4 | 7.2± 0.2 |

This was a blinded study. Each sample was assigned a unique laboratory identification number; for patients from whom more than one sample was collected, the identifier was suffixed with A, B, C, etc. Overlap between pre-transplant and post-transplant samples could only be inferred retrospectively from laboratory identifiers. Using this approach, six of the twelve pre-transplant patients were identified as having corresponding post-transplant samples. A further 22 post-transplant patients provided multiple samples. One patient only provided pre-transplant samples. At least two samples were collected per patient, with up to five samples in some cases. Some patients had multiple samples within a group. In such cases, TTV load values were averaged for analysis. For illustrative purposes, TTV loads that varied within the same group are presented as individual data points in the table. Abbreviations: *TTV,* Torque Teno Virus; *Tx,* Transplant.

**Supplementary Table S2:** The Effect of Patient Group on TTV load relative to Control Cohort

| Group | β | SE | *p* |
| --- | --- | --- | --- |
| Intercept | 3.01 | 0.24 | <.001 |
| CRF | -0.33 | 0.39 | 0.403 |
| Pre-transplant | -0.73 | 0.66 | 0.269 |
| 0-3 months | 0.92 | 0.61 | 0.132 |
| 3-6 months | 2.84 | 0.55 | <0.001 |
| 6-12 months | 2.21 | 0.42 | <0.001 |
| 1 year | 1.77 | 0.46 | <0.001 |
| 2+ years | 1.12 | 0.32 | <0.001 |

*Reference: control cohort. β = difference in log_10_ TTV copies/mL. Robust sandwich standard errors reported.*

TTV loads were significantly higher than in controls from 3 months post-transplantation onward, with the greatest increase observed between 3-6 months post-transplantation.
